# Rectal adenocarcinoma: Ex vivo 9.4T MRI—correlation with histopathologic treatment response to neoadjuvant chemoradiotherapy

**DOI:** 10.1002/cam4.70075

**Published:** 2024-08-01

**Authors:** Zhihui Li, Yuan Yuan, Minglu Liu, Tingting Bo, Xiaolu Ma, Hanqi Wang, Chen Chen, Xiaohui Shi, Hao Wang, Chenguang Bai, Xiang Ni, Chengwei Shao, Yong Lu, Jianping Lu, Fu Shen

**Affiliations:** ^1^ Department of Radiology, Ruijin Hospital Luwan Branch Shanghai Jiaotong University School of Medicine Shanghai China; ^2^ Department of Radiology, Changhai Hospital The Navy Medical University Shanghai China; ^3^ Department of Radiology, Ruijin Hospital Shanghai Jiao Tong University School of Medicine Shanghai China; ^4^ Department of Endocrine and Metabolic Diseases Shanghai Institute of Endocrine and Metabolic Diseases, Ruijin Hospital, Shanghai Jiao Tong University School of Medicine Shanghai China; ^5^ Clinical Neuroscience Center, Ruijin Hospital Luwan Branch Shanghai Jiao Tong University School of Medicine Shanghai China; ^6^ United Imaging Healthcare Shanghai China; ^7^ Department of Colorectal Surgery, Changhai Hospital The Navy Medical University Shanghai China; ^8^ Department of Pathology, Changhai Hospital The Navy Medical University Shanghai China

**Keywords:** ex vivo, magnetic resonance imaging, pathological tumor regression, rectal cancer

## Abstract

**Objectives:**

To determine the imaging details and diagnostic information of the treatment response to neoadjuvant chemoradiotherapy (nCRT) of rectal adenocarcinoma at 9.4T magnetic resonance imaging (MRI) by ex vivo.

**Methods:**

Fifteen cases with locally advanced rectal cancer (LARC) followed by radical surgery after nCRT between September 2022 and February 2023 were recruited. Resected specimens were fixed in a perfluoropolyether‐filled test tube and scanned with a 3.0T and 9.4T MRI system ex vivo. The residual tumor depth and MRI‐based tumor regression grade (TRG) were subjectively assessed and then compared with the pathological findings.

**Results:**

The ex vivo 9.4T T2WI without fat suppression clearly differentiated tumor tissue, fibrosis and normal rectal wall, which clearly corresponded to the pathologic tissues of the rectal specimens. The TRG could be accurately assessed on ex vivo 9.4T images in 13/15 specimens (86.7%), while in 11/15 specimens (73.3%) on ex vivo 3.0T images.

**Conclusion:**

Ex vivo 9.4T MR imaging clearly displayed the components of rectal wall and proved excellent diagnostic performance for evaluating the treatment response to nCRT, which allow radiologists to understand and then assess more accurately the TRG of LARC after nCRT.

## Graphical abstract

MR imaging at 9.4T offers a powerful approach with detailed visualization capabilities for assessing residual tumor invasion and distinguishing between different treatment responses in patients with locally advanced rectal cancer following nCRT.

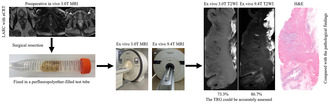

## 1 INTRODUCTION

Locally advanced rectal cancer (LARC) is commonly treated with neoadjuvant chemoradiotherapy (nCRT) combined with total mesorectal resection (TME).^1^, ^2^ Treatment response to nCRT is highly crucial for long‐term prognosis and treatment decision‐making in LARC patients.^3^, ^4^, ^5^ Following nCRT, approximately 15%–27% of patients with LARC achieve pathological complete response (pCR), which is correlated with favorable survival outcome.^4^ Since the “Watch and Wait” approach has been reported for treating LARC patients with a good response to nCRT, nonoperative management (NOM) is considered a promising therapeutic option, with broad acceptance by surgeons and patients.^3^, ^4^, ^5^ However, before NOM is broadly applied clinically, existing challenges need to be addressed, including an accurate selection of NOM‐suitable cases. Therefore, clinical judgments, generally comprising digital rectal examination (DRE), endoscopy, and magnetic resonance imaging (MRI), have been developed for identifying cases with a complete response who are suitable for NOM prior to surgical resection. Yet, the status of residual tumor and pathological tumor regression grade (TRG) can only be accurately identified as a gold standard after radical excision.^6^

Currently, rectal high‐resolution MRI is considered an efficient routine imaging method for the assessment of treatment response to nCRT in patients with LARC.^7^ An MRI‐based tumor regression grading (mrTRG) system was proposed by the Magnetic Resonance Imaging and Rectal Cancer European Equivalence (MERCURY) study group following the principles of five‐category pathological TRG (pTRG) system developed by Dworak et al. and Mandard et al.^6^ This routine preoperative approach evaluated mrTRG after nCRT by discriminating viable tumor signals from fibrosis mainly based on T2WI.

However, conventional high‐resolution MRI at 3.0T is still incapable of resolving the residual tumorous tissue within fibrotic regions and the individual layers of the rectal wall because of limitations in spatial resolution, which can be more inaccurate after nCRT.^8^, ^9^ Moreover, consistency has not been unequivocally demonstrated between mrTRG and pTRG based on the American Joint Committee on Cancer/College of American Pathologists (AJCC/CAP) four‐category TRG classification scheme,^10^, ^11^ with sensitivity and specificity for mrTRG = 1/2 in pCR prediction of only 69.9% and 62.2%, respectively, as reported by a meta‐analysis.^12^ Consequently, it might be limited as a criterion for less aggressive treatment after nCRT.^13^, ^14^ Thus, a powerful approach with detailed visualization capabilities for assessing residual tumor invasion and distinguishing between different treatment responses remains to be developed.

The spatial resolution increases along with the level of magnetic resonance imaging field. 7.0 T MRI scanners have recently been developed for acquiring high‐spatial‐resolution MRI and examined for multiple applications, for example, neuroimaging, cerebrovascular disease, atherosclerosis, prostate diseases, gastric carcinoma, and recently rectal cancer (RC).^15^, ^16^, ^17^, ^18^, ^19^, ^20^, ^21^, ^22^, ^23^ However, there have been no clear research of using the 9.4T MRI system to evaluate the RC ex vivo. In addition, the utility of ultrahigh‐spatial‐resolution MRI for evaluating tumor response to nCRT in RC has not yet been explored. Therefore, in this ex vivo study, we investigated the imaging detail and diagnostic information that can be obtained at 9.4T MRI from excised rectal adenocarcinoma specimens to evaluate tumor regression after nCRT and to compare the diagnostic performance with 3.0T MRI findings.

## 2 METHODS

### 2.1 Study population

**FIGURE 1:**
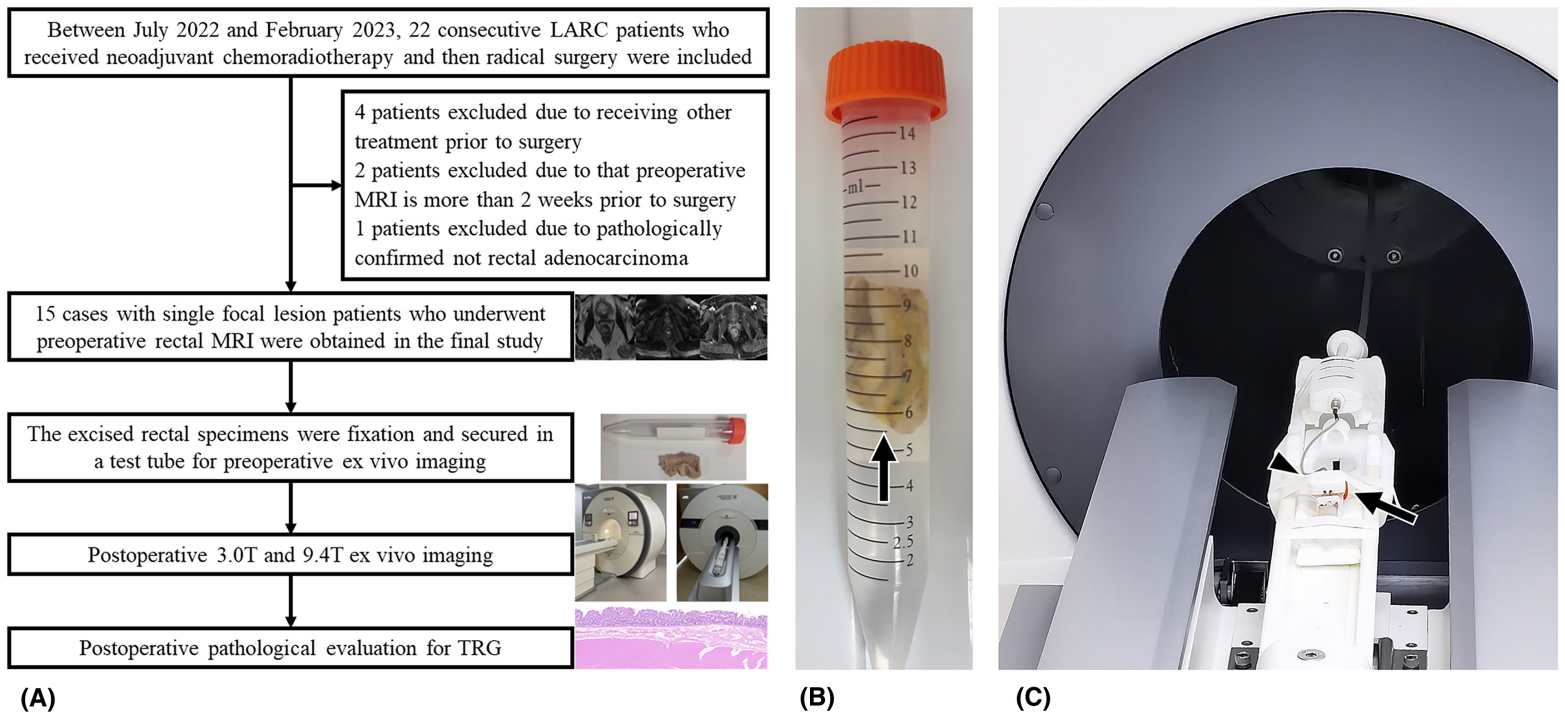
Flow diagram of the study process. (A) The participant selection. (B) The rectal specimen section ex vivo. The excised rectal section (arrow) was set longitudinally along the long axis of the test tube, which filled with FOMBLIN perfluoropolyether. (C) MR scanner and rectal section in tube for imaging. The entire tube (arrow) covered with single loop coil (arrowhead) was examined ex vivo on 9.4T MRI system with small magnet bore size, high magnetic field strength, and strong magnetic field gradients.

This study had approval from the Committee on Ethics of Biomedicine, Changhai Hospital, The Navy Medical University, Shanghai, China, and each patient provided signed informed consent. Between September 2022 and February 2023, 22 surgical specimens of LARC were considered for the study. Inclusion criteria were: (1) underwent preoperative rectal MRI; (2) received standard nCRT, followed by a waiting interval of 4 to 12 weeks and then preoperative MRI; (3) single focal lesion. The exclusion criteria were: (1) received other treatment prior to surgery, *n* = 4; (2) preoperative MRI is more than 2 weeks prior to radical surgery, *n* = 2; (3) pathologically confirmed beyond rectal adenocarcinoma, *n* = 1. The selection criteria are depicted in Figure 1A.

One of the coauthors, Chen Chen, is an employee at the United Imaging Healthcare (UIH), Shanghai, China. Other coauthors who are not employed by UIH had access to all data and information submitted in this article.

### 2.2 Preoperative in vivo MRI

Preoperative rectal MRI was scanned on 3.0T MR systems using a phased array coil. The major sequences comprised oblique‐axial high‐resolution T2WI, axial CE‐T1WI and axial DWI are shown in Table S1; Figure S1.

### 2.3 Specimen preparation

After surgery, the excised rectal specimen was fixated in 10% formalin for 48 h, serving as the interval between surgery and ex vivo imaging. Two experienced pathologists (Chenguang Bai and Xiang Ni, with 24 and 11 years of experience in histopathology) selected the best area for pathologic specimen cutting. Each case selected six optimal sections which were subsequently imaged and pathologically evaluated. The chosen sections were secured in a 15 mL test tube, respectively, which filled with FOMBLIN perfluoropolyether (Solvay Specialty Polymers, Italy) for susceptibility matching and improved magnetic field homogeneity. The specimen was degassed to remove all air bubbles in the sample at 20°C. The excised rectal section was set longitudinally along the long axis of the test tube (Figure 1B).

### 2.4 Postoperative ex vivo image acquisition

The entire tube containing the sample was subsequently imaged ex vivo on two MRI systems (UIH uMR 890 3.0T scanner and UIH uMR 9.4T scanner, Shanghai, China), respectively. The 3.0T scanner utilized a mouse coil and the 9.4T scanner utilized a 12 × 18 mm single loop coil. The tube covered with single loop coil is presented in Figure 1C. The imaging protocol for ex vivo assessment was T2WI in both two above MRI systems. The orientation was set longitudinally along the long axis of the resected segment. The 9.4T ex vivo images were obtained with a voxel volume of 0.08 × 0.08 × 0.7 mm, meanwhile the 3.0T ex vivo images resulted in a voxel volume of 0.29 × 0.29 × 1.0 mm. The mainly used sequence for evaluating residual tumor and TRG was T2WI without fat suppression, consistent with in vivo approach. The detailed parameters are presented in Table 1.

**TABLE 1:** The main sequences protocol and parameters for ex vivo image acquisition.

Scanner	UIH uMR 890	UIH uMR
Magnetic field strength	3.0T	9.4T
Sequence	T2WI	T2WI
Coil	Mouse coil	Single loop coil
Echo train length	13	17
Field of view (mm)	35 × 42	20 × 20
Section thickness (mm)	1.0	0.7
Gap	0	0
Slices	16	6
Matrix	120/144	240 × 240
Repetition time/echo time (ms)	4000/83.64	3000/41.28
Bandwidth (Hz/Pixel)	150	300
Flip angle (°)	120	180
Number of averages	6	16
Acquisition time	33 min 48 s	12 min 03 s

*Note*: UIH, United Imaging Healthcare, Shanghai, China.

### 2.5 Objective image evaluation

Two radiologists (R1 and R2) evaluated the signal‐to‐noise ratio (SNR) and contrast‐to‐noise ratio (CNR) independently on T2WI in all sections. R1 (Zhihui Li) is a radiologist with 11 years of experience in assessing MRI. R2 (Yuan Yuan) has 13 years of experience in assessing MRI. Three regions of interest (ROIs) were delineated with a circle (with diameter of at least 1 mm) on same slice, as submucosa, propria muscle, and background. The signal intensities of ROIs were measured three times, then averaged the results to calculate SNR and CNR.

The definitions of SNR and CNR were as follows:

$$\mathrm{SNR}={\mathrm{SI}}_{\text{submucosa}}/\sigma_{\text{background}}.$$

$$\mathrm{CNR}=\left({\mathrm{SI}}_{\text{submucosa}}-{\mathrm{SI}}_{\text{propria muscle}}\right)/\sigma_{\text{background}}.$$

where SI and *σ* are mean signal intensity and standard deviation of the ROI, respectively.

### 2.6 Subjective image evaluation

In the subjective assessment of image quality, R1 and R2 conducted independent reviews of the lesion display performance and overall image quality for the two sets of ex vivo images, utilizing a 5‐point scale, with 1 to 5 reflecting images not appropriate for diagnosis, with poor quality but still interpretable, with acceptable quality, with good quality, and with excellent quality, respectively. Criteria included a nuanced evaluation of detailed display within the lesion, the outer boundary of the lesion, as well as considerations for artifacts and deformations.

### 2.7 MRI performance and diagnostic value

All in vivo and ex vivo MR images were evaluated for analyzing residual tumor invasion and treatment response, respectively. We proposed a four‐category ex vivo mrTRG (evmrTRG) system to investigate its diagnostic value for evaluating TRG and consistency with the AJCC/CAP system, especially in cases with pCR. The residual tumor invasion, mrTRG and evmrTRG were independently evaluated by R1 and R2. In case of discrepancy, R3 (Fu Shen), a senior investigator with 15 years of experience in radiology specialized in rectal MRI, was involved for settlement. All six sections were evaluated for each case and then rated final evmrTRG. All readers were blinded to the pathological findings.

**FIGURE 2:**
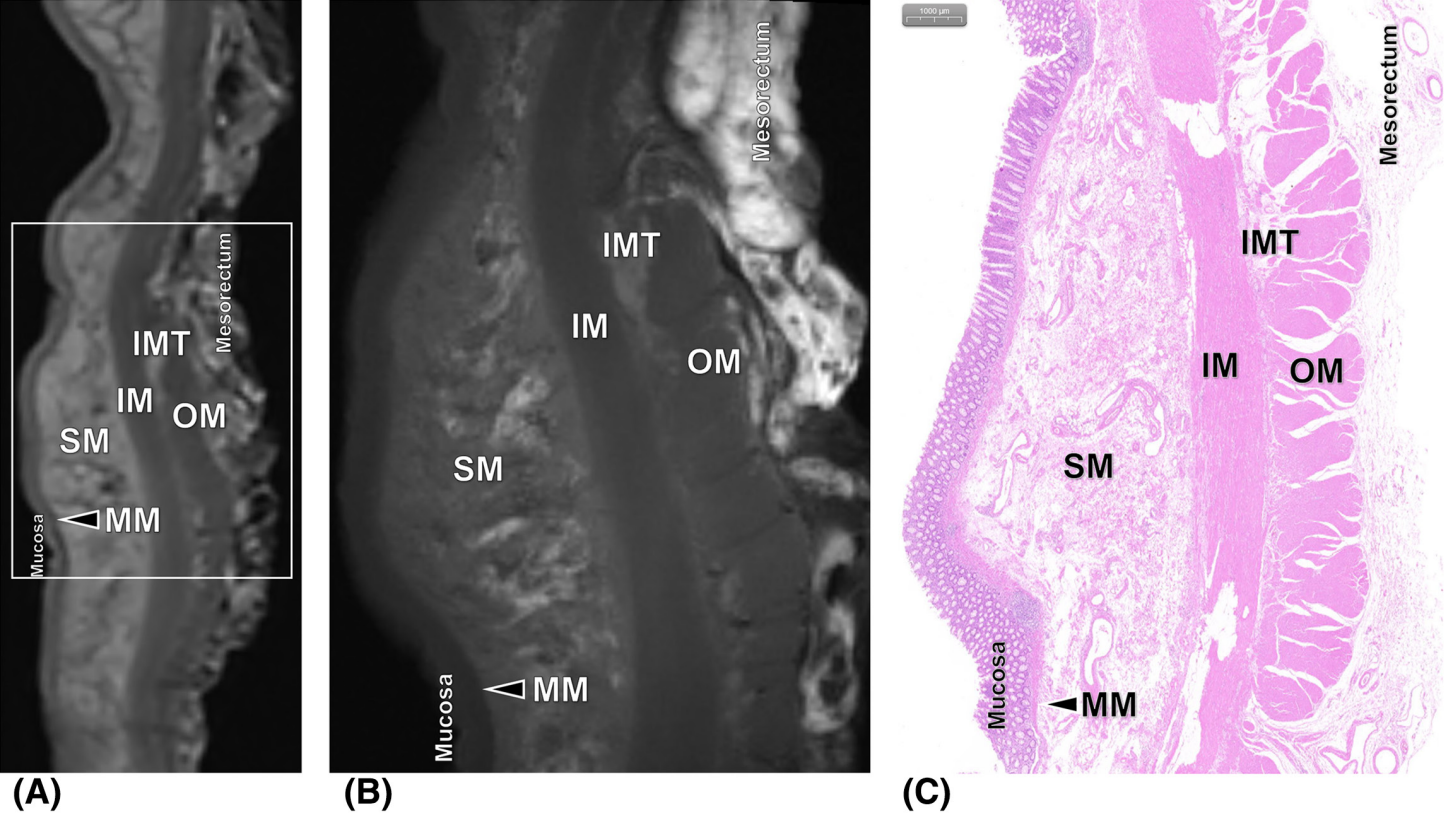
Images of the normal rectal wall obtained from the normal portion of the surgical specimen. (A) Ex vivo T2‐weighted 3.0T MR image and (B) ex vivo T2‐weighted 9.4T MR image without fat suppression clearly show that the normal rectal wall consists of the following seven layers: mucosa, muscularis mucosae (MM), submucosa (SM), inner muscle (IM), intermuscular tissue (IMT), outer muscle (OM), and mesorectum. (C) Histopathologic slice of the normal rectal wall shows the mucosa, MM, SM, IM, IMT, OM, and mesorectum (hematoxylin–eosin stain, ×20).

To improve the applicability and stability of the ex vivo MR evaluation criteria, one normal rectal specimen obtained from the normal portion of a surgical specimen and one RC specimen without nCRT were enrolled as a preliminary baseline analysis. The baseline ex vivo MR images were reviewed for the normal rectal wall presence, signal intensity (SI) of each layer of the rectal wall (Figure 2), and the SIs of tumor were also analyzed (Figure S2).

### 2.8 Pathological evaluation

All specimens were prepared routinely and 10% formalin fixed, at last slices of paraffin‐embedded biopsy were produced. All excised rectal sections underwent hematoxylin–eosin (H&E) staining. The pathological evaluation served as the reference standard after discussion by two experienced pathologists blinded to MRI findings.

The surgical samples were examined as proposed by Quirke and Dixon,^24^ based on the National Comprehensive Cancer Network and American Joint Committee on cancer (AJCC Cancer Staging Manual, 8th edition) staging system; response grades were determined with the four‐category pTRG system^25^ as follows: TRG 0, no remaining viable cancer cells (complete response); TRG 1, only small cluster or single cancer cells remaining (moderate response); TRG 2, residual cancer remaining, but with predominant fibrosis (minimal response); and TRG 3, minimal or no tumor kill with extensive residual cancer (poor response).^25^ If one case was described as TRG 0 or 1, pathologists re‐examined the entire tumor bed of the specimen. pTRG = 0 was identified as pCR (ypT0N0M0).

### 2.9 Statistical analysis

SPSS (version 22.0, Inc., Chicago, IL, USA) and MedCalc Statistical software (version 13.0.0.0, MedCalc Software, Mariakerke, Belgium) were used to perform statistical analyses. The Kolmogorov–Smirnov test was used to check for the normality of continuous variables. Continuous variates were assessed for normality by the Kolmogorov–Smirnov test; those with normal distribution were expressed as mean ± standard deviation. Categorical variates were presented as percentage and compared by the Chi‐squared test or Fisher's exact test. Both signal‐ (SNR) and contrast‐ (CNR) to‐noise ratios adhered to normal distributions. The comparison of SNR and CNR between ex vivo 3.0T and 9.4T T2W images was conducted using paired‐sample *t*‐tests. Intraclass correlation coefficient (ICC) was performed for SNR and CNR between Readers. The Paired Wilcoxon test was used to assess the comparison of image quality scores for the two sets of ex vivo images. The Weighted kappa coefficient was determined to evaluate the concordance rate between subjective evaluation and pTRG. Accuracy rates in diagnosing pTRG were calculated and compared between ex vivo 3.0T and 9.4T using Fisher's exact test. *p* < 0.05 was deemed statistically significant.

## 3 RESULTS

### 3.1 Patient characteristics

In the final analysis, a total of 15 cases involving rectal adenocarcinoma were enrolled in the final analysis of 6*15 sections. There were 7 cases identified as pCR (pTRG = 0) according to the pathologic interpretations by two pathologists in consensus. Meanwhile, TRG = 1, TRG = 2 and TRG = 3 was 1, 4 and 3 cases, respectively. The characteristics of patients are listed in Table S2.

### 3.2 Ex vivo MR images presentation

The SIs of the rectal wall and lesion on ex vivo T2WI without fat suppression are shown in Figure 2, Table 2 and Figure S2. On ex vivo T2W images without fat suppression, the normal rectal wall was presented as consisting of the following seven layers: (1) mucosa, (2) muscularis mucosae (MM), (3) submucosa, (4) inner muscle, (5) intermuscular tissue, (6) outer muscle, and (7) mesorectum. The submucosa had high to intermediate SI values, with the presence of blood vessels which appeared with low SI. The SI of tumor was low to intermediate, lower than that of the mesorectum and submucosa, higher than that of the MM, inner and outer muscle. The area with treatment associated fibrotic changes had relatively low SI in all rectal layers. These SI presentations of ex vivo 9.4T T2W images were all found to clearly correspond to the tissues observed in the histopathologic slices. However, unobvious distinction of small amount of residual tumor and fibrosis area in 4 cases was observed on ex vivo 3.0T T2W images. The SIs of the rectal wall and lesion on ex vivo T2W images with fat suppression are shown in Table S3.

**TABLE 2:** Signal intensities on ex vivo T2‐weighted images without fat suppression.

Tissue	Ex vivo T2WI without fat suppression	
	3.0T	9.4T
Mucosa	Intermediate	Low to intermediate
Muscularis mucosae	Low to intermediate	Low
Submucosa	High	High to intermediate
Inner muscle	Low to intermediate	Low
Intermuscular tissue	High to intermediate	Intermediate
Outer muscle	Low to intermediate	Low
Mesorectum	High	High
Fibrosis	Low	Low
Carcinoma	Low to intermediate	Low to intermediate
Blood vessel	Low	Low

### 3.3 Objective image evaluation

On ex vivo 3.0T and 9.4T MRI images without fat suppression, the mean value of CNR derived from 9.4T MRI images was significantly higher than 3.0T images (77.310 ± 48.897 vs. 34.290 ± 29.137, *p* < 0.0001). The difference of SNR between them was not statistically significant (129.990 ± 79.780 vs. 145.928 ± 49.489, *p* > 0.05). The ICCs of CNR of ex vivo 3.0T and 9.4T MRI images between two radiologists were 0.961 and 0.975, had excellent agreement (Table S4). Meanwhile, on ex vivo T2W images with fat suppression, the mean CNR and SNR of ex vivo 9.4T MRI images were 8.489 ± 5.371 and 60.159 ± 12.395, lower than those of 3.0T images with 44.388 ± 19.830 and 203.476 ± 32.696 (*p* < 0.0001). Therefore, we mainly used T2WI without fat suppression sequence for subsequent evaluation, consistence with preoperative in vivo approach.

### 3.4 Subjective image evaluation

Regarding the subjective assessment of image quality, the scores assigned to the ex vivo 9.4T image set exhibited a statistically significant increase compared to those of the ex vivo 3.0T for both radiologists (5 [4, 5], 5 [4, 5] and 4 [3, 4], 4 [4], *p* < 0.0001).

### 3.5 Evaluation of residual tumor invasion

The comparison of MRI and histopathologic findings in evaluation of the residual tumor invasion is presented in Table S5. In 8 cases (53.3%, 8/15), the preoperative rectal MRI accurately diagnosed the residual depth of invasion of the rectal wall according to the ypT stage (“yp” denote pathologic staging following neoadjuvant therapy). Meanwhile, the accuracies of ex vivo 3.0T and 9.4T MRI in evaluation of residual tumor invasion were 80% (12/15) and 100% (15/15), respectively, significantly higher than the in vivo MRI approach (*p* = 0.0086).

### 3.6 Assessment of tumor regression

The subjective analysis of in vivo 3.0T MRI showed that the mrTRG were associated with the pathologic findings in evaluation of the TRG indirectly (Table S6; Figure S1), only one case of TRG 0 was diagnosis correctly as mrTRG 1 by the definition of MERCURY group (14.3%, 1/7 cases).

**FIGURE 3:**
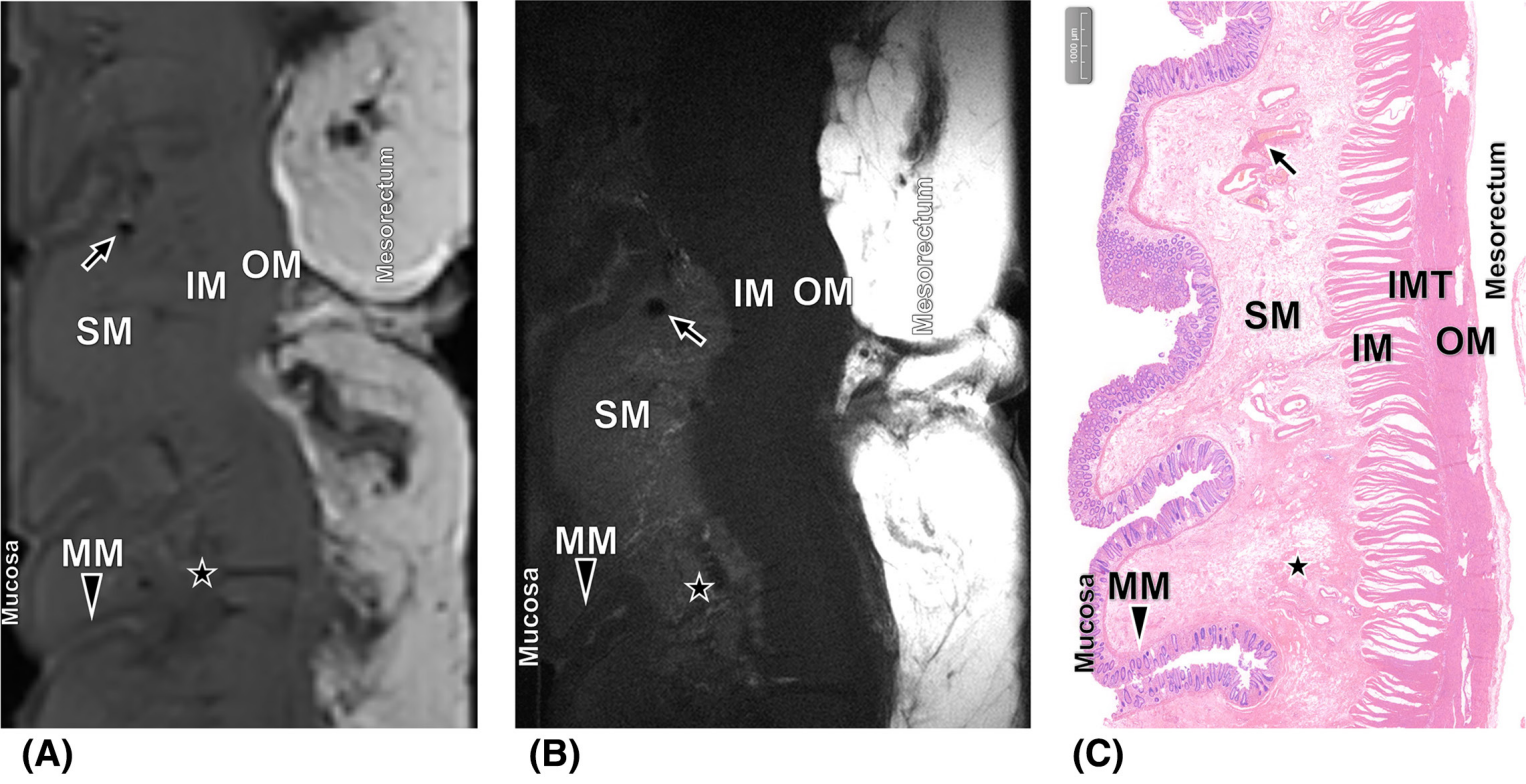
Images of the complete response performance obtained from the patient who pathologically diagnosed as TRG 0. (A) Ex vivo T2‐weighted 3.0T MR image and (B) ex vivo T2‐weighted 9.4T MR image clearly show no remaining viable residual tumor with an area of low SI in the submucosa (SM), diagnosed as evmrTRG 0. It is noteworthy that, this probably because of nCRT associated fibrotic changes and appear with low SI on T2W images (star). The normal SM had high to intermediate SI values on T2W images, with the presence of blood vessels which appeared with low SI (arrow). (C) Corresponding histopathologic slice shows complete response that has no residual tumor and slight fibrotic changes in the SM (hematoxylin–eosin stain, ×20).

**FIGURE 4:**
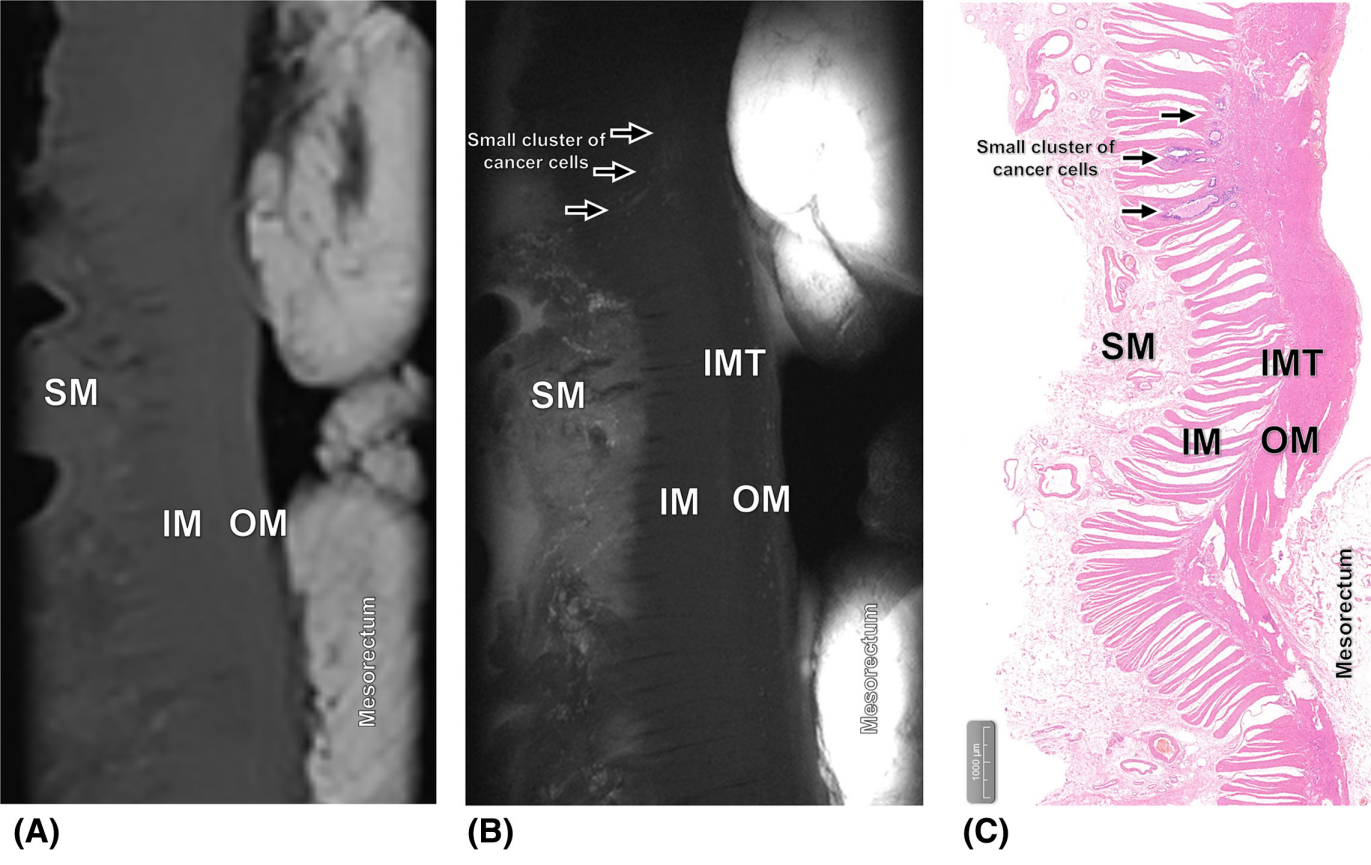
Images of the significant response performance obtained from the patient who pathologically diagnosed as TRG 1. (A) Ex vivo T2‐weighted 3.0T MR image shows no obvious residual tumor, recognized as evmrTRG 0. (B) Ex vivo T2‐weighted 9.4T MR image shows a small cluster of cancer cells scattered in the inner muscle (IM) and intermuscular tissue (IMT) and appear with low to intermediate SI (arrow), diagnosed as evmrTRG 1. (C) Corresponding histopathologic slice shows significant response that has only small clusters of cancer cells in the IM and IMT (hematoxylin–eosin stain, ×20).

**FIGURE 5:**
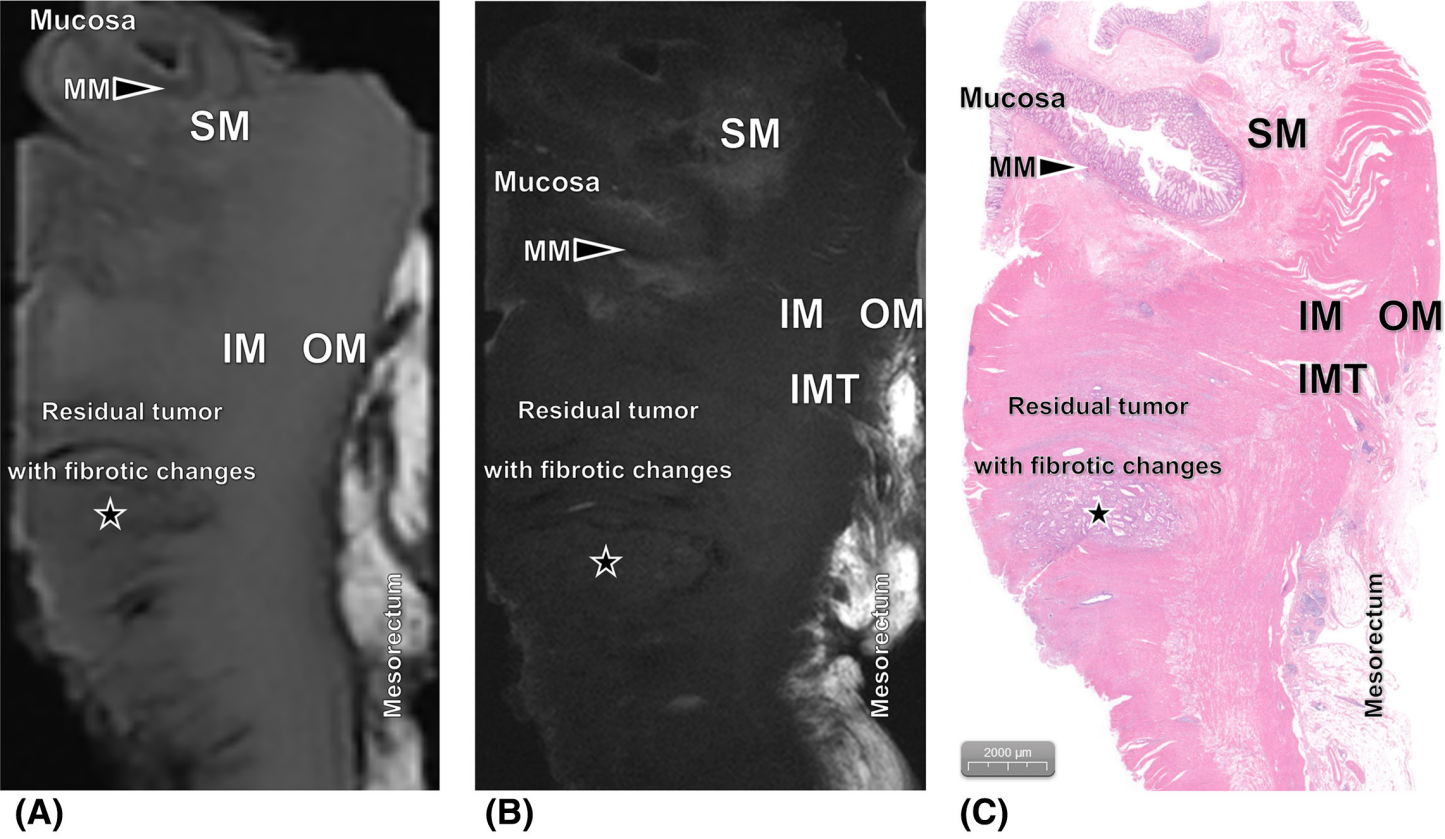
Images of the poor response performance obtained from the patient who pathologically diagnosed as TRG 2. (A) Ex vivo T2‐weighted 3.0T MR image shows residual tumor with slight fibrotic changes (star) which has unclear hierarchical structure between submucosa (SM) and outer muscle (OM) layers, recognized as evmrTRG 3, while (B) ex vivo T2‐weighted 9.4T MR image show presence of residual tumor with treatment associated fibrotic changes reduced SI of the area of SM (star), diagnosed as evmrTRG 2. (C) Corresponding histopathologic slice shows poor response that has presence of residual tumor with obvious fibrosis in the SM, IM and IMT (hematoxylin–eosin stain, ×10).

**FIGURE 6:**
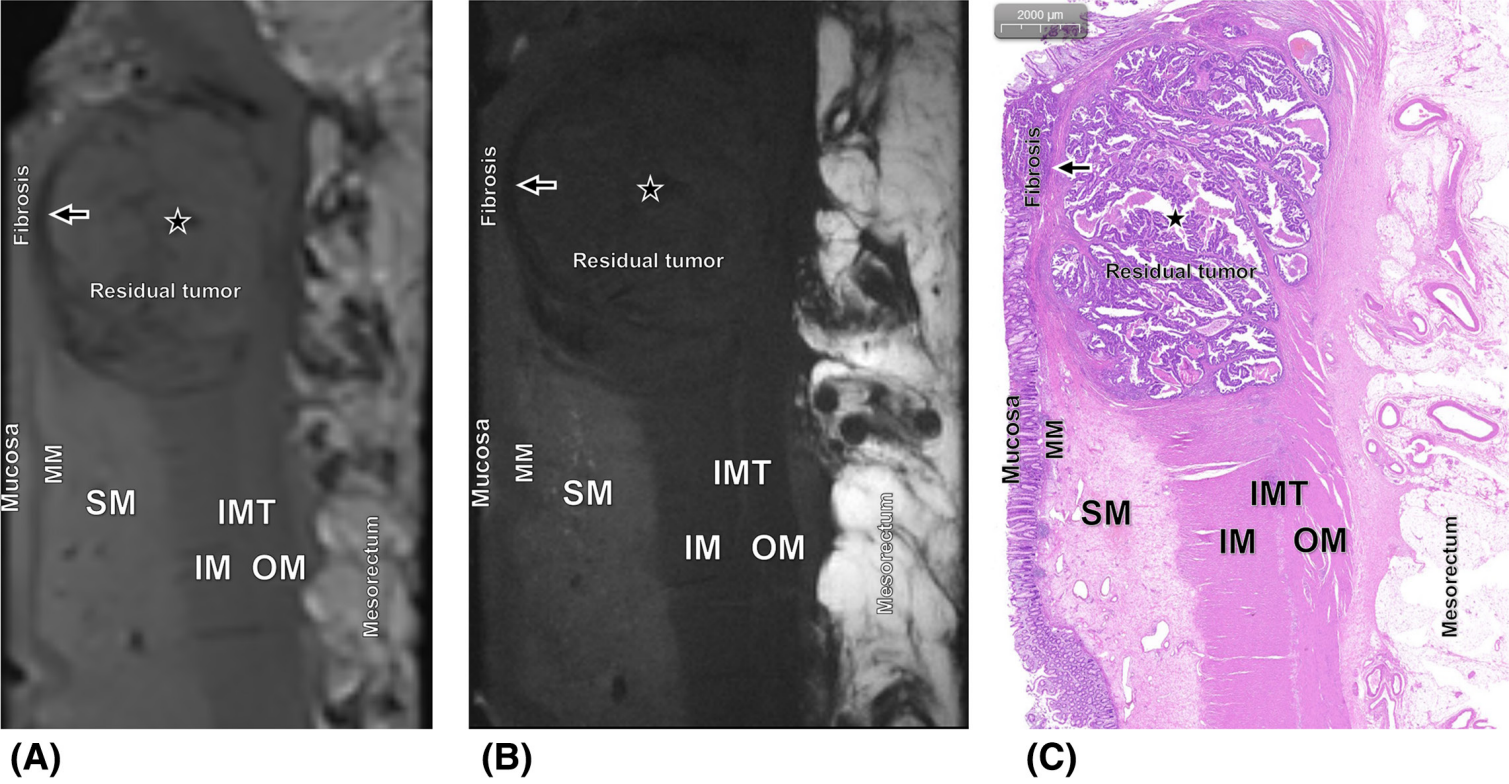
Images of the no response performance obtained from the patient who pathologically diagnosed as TRG 3. (A) Ex vivo T2‐weighted 3.0T MR image and (B) ex vivo T2‐weighted 9.4T MR image show presence of extensive residual tumor (star) with a small amount of fibrosis (arrow), diagnosed as evmrTRG 3. (C) Corresponding histopathologic slice shows no response that has obvious residual tumor with slight fibrosis between the mucosa and outer muscle (OM) (hematoxylin–eosin stain, ×10).

After consensus interpretation, the ex vivo 3.0T and 9.4T MR images enabled correct diagnosis of pTRG in 11/15 (73.3%) and 13/15 (86.7%) RC cases that were studied (Figures 3, 4, 5, 6). Given the relatively modest sample size, the difference in accuracies between them did not achieve statistical significance (Fisher's exact test, *p* = 0.651). The Kappa values were 0.926 and 0.960, respectively (Tables 3 and 4). In two specimens, discrepancy occurred between the 3.0T and 9.4T evmrTRG. One specimen of TRG 1 was recognized as evmrTRG 0 on ex vivo 3.0T, but diagnosed correctly on 9.4T (Figure 4). Another case with TRG 2 was identified as evmrTRG 3 on ex vivo 3.0T, but it was accurate on 9.4T (Figure 5).

**TABLE 3:** Comparison of ex vivo 3.0T MR images and histopathologic findings in evaluation of the TRG.

Ex vivo	Pathological TRG			
3.0T MR images	pTRG 0 (*n* = 7)	pTRG 1 (*n* = 1)	pTRG 2 (*n* = 4)	pTRG 3 (*n* = 3)
evmrTRG 0	7	1	0	0
evmrTRG 1	0	0	0	0
evmrTRG 2	0	0	1	0
evmrTRG 3	0	0	3	3

*Note*: Total accuracy = 11/15 (73.3%), kappa = 0.926.

Abbreviation: *evmrTRG*, ex vivo MRI‐based tumor regression grading.

**TABLE 4:** Comparison of ex vivo 9.4T MR images and histopathologic findings in evaluation of the TRG.

Ex vivo	Pathological TRG			
9.4T MR images	pTRG 0 (*n* = 7)	pTRG 1 (*n* = 1)	pTRG 2 (*n* = 4)	pTRG 3 (*n* = 3)
evmrTRG 0	7	0	0	0
evmrTRG 1	0	1	0	0
evmrTRG 2	0	0	2	0
evmrTRG 3	0	0	2	3

*Note*: Total accuracy = 13/15 (86.7%), kappa = 0.960.

Abbreviation: *evmrTRG*, ex vivo MRI‐based tumor regression grading.

For comparing the pCR with MRI findings, the evmrTRG reached a good performance (100%, both 7/7), significantly higher than the mrTRG approach of 14.3% (*p* = 0.0002). In addition, for assessment of near‐pCR (identified as pTRG = 0–1), there was no significant difference among the three approaches (Table S7).

## 4 DISCUSSION

This study demonstrated the feasibility of 9.4T MRI for RC specimens ex vivo for assessing the tumor regression after nCRT. Using the T2‐weighted imaging sequence without fat suppressed, our ex vivo 9.4T MR protocol provided improved CNR and high resolution for rectal wall layers, allowing reasonably accurate assessment of residual tumor invasion and the tumor regression grade in RC specimens. The diagnostic performance of both ex vivo 3.0T and 9.4T was superior to that of in vivo 3.0T. Furthermore, the diagnostic performance at 9.4T may offer an advantage over ex vivo 3.0T for evaluating the treatment response to nCRT in rectal adenocarcinoma (86.7% vs. 73.3%).

Recent randomized trials had also demonstrated that the oncologic outcomes of patients undergoing Nonoperative Management (NOM) were not statistically different from those undergoing Total Mesorectal Excision (TME), but NOM resulted in fewer postoperative complications.^12^ The administration of NOM relies on identifying the response to neoadjuvant therapy according to clinical assessment. However, the status of residual tumor and Pathological Tumor Regression Grade (TRG) can only be accurately determined as a gold standard after radical excision. Nowadays, identifying the tumor response to nCRT for LARC based on clinical assessment is often a challenge. The MERCURY study proposed the mrTRG system that closely resembled the five‐category pTRG.^6^ Despite similarities with pTRG principles, whether the conventional 5‐category mrTRG system could mimic the pTRG classification scheme is debatable; since the mrTRG system is considered suboptimal, it may be merely a criterion for less aggressive therapy following nCRT.^26^, ^27^, ^28^, ^29^, ^30^, ^31^ Here, referring to the AJCC/CAP 4‐category TRG protocol, a 4‐category evmrTRG system was developed considering ex vivo MRI, and its diagnostic performance and consistency with the pTRG system were examined, especially in patients with pCR.

The overt display of rectal wall layers and tumors on T2‐weighted scans might result from the alternating cellularity levels of the tissues causing marked differences in the T2 relaxation times of the layers.^32^ In cases responding to neoadjuvant therapy, responsive tumors are substituted by dense fibrosis and show low SI on T2‐weighed scans.^12^, ^31^, ^32^, ^33^ However, in LARC patients after nCRT, a small cluster of cancer cells scattered in the large amount of neoadjuvant therapy induces fibrosis may not seem as obvious as postoperative pathologic findings. Thus, tissue differentiation between slight residual tumors and fibrosis represents an important challenge for clinical MRI, which routinely uses a magnetic field of 1.5 or 3.0T.^12^, ^32^

Our previous study developed a modified MRI‐based split scar sign (mrSSS) score for accurate identification of cases with pCR based on post‐nCRT MRI.^31^ Our hypothesis is that the split scar sign features an inner, regular, and markedly T2‐hypointense layer that corresponds to the fibrotic submucosa, either covered by intact mucosa or denuded. The outer T2‐hypointense layer reflects the fibrotic muscularis propria. However, this mrSSS score system describes a simple visual pattern on routine post‐nCRT T2WI; it not only lacks enough sensitivity but also lacks in‐depth mechanism research.^31^ Thus, we aim to develop a powerful approach with detailed visualization capabilities for distinguishing different treatment responses and helping clinicians better understand the split scar sign.

Surgical samples can be assessed using preclinical MR systems with reduced magnet bore size, elevated magnetic field strength, and heftier magnetic field gradients. Employing a dedicated ex vivo MRI protocol to examine rectal samples, high spatial resolution images can display all rectal layers. Therefore, by distinguishing signal differences, ultrahigh magnetic field MR imaging may make it possible to depict subtle pathological changes after neoadjuvant treatment. Thanks to an extremely high spatial resolution for different tissues, 7.0 T ex vivo MRI is very accurate in the diagnosis of gastric and rectal diseases.^21^, ^22^, ^23^ Here, MRI was carried out at 9.4T, with very small voxel volume (0.08 × 0.08 × 0.7 mm). By depicting subtle pathological changes, the proposed MR method helped obtain high spatial resolution images and high CNR, which may make it possible to differentiate neoadjuvant chemotherapy‐induced fibrosis from residual tumor tissue and rectal wall layers. As a result, the performance of ex vivo 9.4T MRI is advantageous when compared with ex vivo 3.0T MRI and the currently applied convention of employing T2‐weighted imaging at 3.0T.

There were some limitations in this study. First, our data showed that 9.4T T2W images of good quality enabled correct diagnosis of the ypT stage in all 15 cases (100%) and correct diagnosis of pTRG in 13/15 (86.7%) studied cases. However, since there was disagreement in two cases that was recognized as evmrTRG 3, but pathological diagnosis as pTRG 2, it may be due to subjective error and the limited range of section sampling when evaluating the percentage of residual tumor with inconspicuous fibrosis, meanwhile these issues also constitute a limitation in postoperative pathological evaluation.

Second, limited ex vivo sequences were performed in our study. Due to this relatively novel preclinical MR system, many sequences have not yet been optimized, such as DWI and T1 mapping. Thus, majorization of novel technologies is required for a more technically feasible utilization in clinic, and the diagnostic performance of this ex vivo approach remains to be further studied. Moreover, being a single‐center study, only 15 resected specimens were enrolled and selection biases may have influenced our findings. Thus, for further validation and enhancing the generalizability of our results, larger datasets and multi‐center studies are necessary.

Finally, in our study the samples were assessed ex vivo by imaging upon formalin fixation. When the specimen was fresh, Imai and colleagues^34^ reported slightly higher SI for the muscularis propria on T2WI of the colorectal wall, with slightly reduced contrast in the submucosa versus the muscularis propria. Whether the above findings are applicable to fresh samples is unknown.

## 5 CONCLUSION

In conclusion, we have demonstrated that the T2WI without fat suppression on 9.4T magnetic resonance imaging for ex vivo rectal specimen has improved CNR and ultra‐high spatial resolution. This novel technology was closely related to the pathological findings and may prove valuable in evaluation of the tumor regression, which could possibly be a viable option in assessment of the treatment response to nCRT in the patients with LARC.

## AUTHOR CONTRIBUTIONS

**Zhihui Li:** Conceptualization (equal); investigation (equal); methodology (equal); writing – original draft (equal). **Yuan Yuan:** Data curation (equal); formal analysis (equal); funding acquisition (equal); writing – original draft (equal). **Minglu Liu:** Validation (equal); visualization (equal); writing – original draft (equal). **Tingting Bo:** Data curation (equal); methodology (lead); resources (equal); validation (equal); visualization (equal); writing – original draft (equal). **Xiaolu Ma:** Data curation (equal); methodology (equal); software (equal). **Hanqi Wang:** Data curation (equal); formal analysis (equal); software (equal). **Chen Chen:** Data curation (equal); investigation (equal); methodology (equal). **Xiaohui Shi:** Data curation (equal); formal analysis (equal); funding acquisition (equal). **Hao Wang:** Resources (equal); supervision (equal); validation (equal); visualization (equal). **Chenguang Bai:** Investigation (equal); methodology (equal); validation (equal); visualization (equal). **Xiang Ni:** Data curation (equal); investigation (equal); methodology (equal). **Chengwei Shao:** Funding acquisition (equal); investigation (equal); resources (equal); supervision (equal); validation (equal); visualization (equal). **Yong Lu:** Conceptualization (equal); funding acquisition (equal); project administration (equal); supervision (equal); visualization (equal); writing – review and editing (equal). **Jianping Lu:** Investigation (equal); project administration (equal); supervision (equal); visualization (equal); writing – review and editing (equal). **Fu Shen:** Conceptualization (lead); data curation (lead); formal analysis (lead); funding acquisition (equal); investigation (equal); methodology (lead); project administration (lead); supervision (equal); validation (equal); visualization (equal); writing – original draft (equal); writing – review and editing (equal).

## FUNDING INFORMATION

This research was financially supported by National Natural Science Foundation of China (82171891), Scientific research program of Shanghai municipal science and technology commission (21ZR1439800), Shanghai International Cooperation Project (22490713400), Foundation of Shanghai Municipal Health Commission (202240204), “Yi Yuan Xin Xing” young medical talents funding project of Shanghai (project's number: N/A) and the Guhai Project of Changhai hospital (GH145‐09).

## CONFLICT OF INTEREST STATEMENT

The authors declare no conflict of interest.

## ETHICS STATEMENT

This study was approved by the ethics committee of the Shanghai Changhai Hospital, Naval Medical University (IRB Approval No. B2022‐013).

## Supporting information


Data S1.

## Data Availability

The datasets used and/or analyzed in the current study are available from the corresponding author on reasonable request.
