# Supplementary material for: Rectal adenocarcinoma: Ex vivo 9.4T MRI—correlation with histopathologic treatment response to neoadjuvant chemoradiotherapy
Source: Cancer Med. 2024 Aug 1;13(15):e70075. doi: 10.1002/cam4.70075 (PMC11293138; doi:10.1002/cam4.70075)
Supplement: Supplementary file 1 — Data S1. [file CAM4-13-e70075-s001.docx]

**Supplemental Table 1.** **Main sequences, protocols, and parameters of in vivo rectal MRI**

| **Scanner** | **GE**  **Discovery 750w** | **SIEMENS**  **Magnetom Skyra** |
| --- | --- | --- |
| **Magnetic field strength** | 3.0T | 3.0T |
| **Oblique axial T2WI** |  |  |
| Echo train length | 32 | 16 |
| Field of view (mm) | 200×200 | 180×180 |
| Section thickness (mm) | 4 | 3 |
| Matrix | 352×352 | 320×320 |
| TR/TE (ms) | 6538/116 | 4000/108 |
| Bandwidth (kHz) | 62.5 | 108/Pixel |
| Flip angle (°) | 110 | 160 |
| **Axial contrast enhanced T1WI** |  |  |
| Field of view (mm) | 340×340 | 300×243.9 |
| Section thickness (mm) | 4 | 4.5 |
| Matrix | 256×224 | 288×201.6 |
| TR/TE (ms) | 4/Minimum | 5.9/2.5 |
| Bandwidth (kHz) | 142.9 | 390/pixel |
| Flip angle (°) | 12 | 9 |
| **Axial DWI** |  |  |
| Field of view (mm) | 320×256 | 380×380 |
| Section thickness (mm) | 6 | 5 |
| Matrix | 128×128 | 150×150 |
| TR/TE (ms) | 2840/Minimum | 6300/89 |
| b values (s/mm^2^) | 0, 1000 | 0, 1000 |

TR/TE: Repetition time/echo time; DWI: diffusion-weighted imaging, *b* values (averages) = 0 (1) and 1000 (3) s/mm^2^; T1WI: T1-weighted imaging; T2WI: T2-weighted imaging.

**Supplemental Table 2. Patient characteristics and pathological outcomes**

| **Characteristics** | **All**  ***n* = 15** |
| --- | --- |
| Gender (male/female) | 10/5 |
| Age (years) ^*^ | 59.0 (48.5, 66.5) |
| BMI (kg/m^2^) ^*^ | 23.9 (22.7, 26.0) |
| Height of tumor ^*^ (cm) | 5.2 (4.25, 9.35) |
| MR T stage ^**^ |  |
| T1-2 | 4 |
| T3-4 | 11 |
| MR N stage ^**^ |  |
| N0 | 7 |
| N1-2 | 8 |
| MRF ^**^ |  |
| Negative | 13 |
| Positive | 2 |
| EMVI ^**^ |  |
| Negative | 6 |
| Positive | 9 |
| Differentiation |  |
| Well-Moderate | 13 |
| Poor | 2 |
| Invasion of the resection margin | 0 |
| Pathological TRG ^#^ |  |
| 0 | 7 |
| 1 | 1 |
| 2 | 4 |
| 3 | 3 |
| ypT stage |  |
| T0 | 7 |
| T1 | 0 |
| T2 | 2 |
| T3 | 6 |
| T4 | 0 |
| ypN stage |  |
| N0 | 9 |
| N1 | 4 |
| N2 | 2 |
| Mismatch repair (MMR) status |  |
| proficient in MMR | 15 |
| deficiency in MMR | 0 |
| Pre-nCRT CEA |  |
| <5 ng/ml | 5 |
| >= 5ng/ml | 10 |
| Pre-nCRT CA19-9 |  |
| < 37U/ml | 9 |
| >= 37U/ml | 6 |
| The interval between nCRT and preoperative MRI ^*^ (week) | 8 (6.25, 8.75) |

^*^ Median (IQR).

^#^ *TRG*, tumor regression grading, referring to the AJCC/CAP TRG category.

“*yp*” denote pathologic staging following neoadjuvant therapy.

*BMI*, body mass index; *CEA*, carcinoembryonic antigen; *CA19-9*, carbohydrate antigen 19-9;

*MRF*, mesorectal fascia; *EMVI*, extramural venous invasion.

^**^ Pre-nCRT baseline MRI.

**Supplemental Table 3. Signal intensities on ex vivo T2-weighted images** **with fat suppression**

| **Tissue** | **ex vivo T2WI** **with fat suppression** | |
| --- | --- | --- |
|  | **3.0T** | **9.4T** |
| Mucosa | Intermediate | Intermediate |
| Muscularis mucosae | Low | Low |
| Submucosa | High | High-intermediate |
| Inner muscle | Intermediate | Intermediate |
| Intermuscular tissue | High | High-intermediate |
| Outer muscle | Intermediate | Intermediate |
| Mesorectum | Low | Low |
| Fibrosis | Low | Low |
| Carcinoma | Low-intermediate | Low-intermediate |
| Blood vessel | Low | Low |

**Supplemental Table 4. SNR and CNR evaluation between** **ex vivo 3.0T and 9.4T MRI**

|  |  | **ex vivo T2WI without fat suppression** | |  |
| --- | --- | --- | --- | --- |
|  |  | **3.0T** | **9.4T** | ***p* value** |
| **R1** | **SNR** | 156.366±75.845 | 150.116±77.336 | 0.588 |
|  | **CNR** | 27.480±27.542 | 73.321±59.077 | <0.0001 |
| **R2** | **SNR** | 105.400±46.044 | 105.133±45.868 | 0.916 |
|  | **CNR** | 42.736±33.580 | 78.053±67.344 | <0.0001 |
| **Mean** | **SNR** | 145.928±49.489 | 129.990±79.780 | 0.258 |
|  | **CNR** | 34.290±29.137 | 77.310±48.897 | <0.0001 |
| **ICC (95% CI) ^*^** | **SNR** | 0.615 (0.395-0.768) | 0.598 (0.372-0.757) |  |
|  | **CNR** | 0.961 (0.930-0.978) | 0.975 (0.955-0.986) |  |

Data are shown as the Mean ± SD.

^*^ *ICC*, intraclass correlation coefficient, compared between R1 and R2.

**Supplemental Table 5.** **Accuracies of evaluating residual tumor invasion**

| **MR images** | **ypT stage ^*^** | | | | |
| --- | --- | --- | --- | --- | --- |
|  | ypT0  (*n* = 7) | ypT1  (*n* = 0) | ypT2  (*n* = 2) | ypT3  (*n* = 6) | All  (*n* = 15) |
| In vivo 3.0T | 1/7 | / | 1/2 | 6/6 | 8/15 |
| Ex vivo 3.0T | 7/7 | / | 0/2 | 5/6 | 12/15 |
| Ex vivo 9.4T | 7/7 | / | 2/2 | 6/6 | 15/15 |

^*^ “*yp*” denote pathologic staging following neoadjuvant therapy.

**Supplemental Table 6.** **Comparison of in vivo MR images and** **pathologic findings for TRG evaluation**

| **In vivo** | **Pathological TRG** | | | |
| --- | --- | --- | --- | --- |
| mrTRG ^*^ | pTRG 0  (*n* = 7) | pTRG 1  (*n* = 1) | pTRG 2  (*n* = 4) | pTRG 3  (*n* = 3) |
| mrTRG 1 | 1 | 1 | 0 | 0 |
| mrTRG 2 | 4 | 0 | 1 | 0 |
| mrTRG 3 | 2 | 0 | 3 | 1 |
| mrTRG 4 | 0 | 0 | 0 | 2 |
| mrTRG 5 | 0 | 0 | 0 | 0 |

^*^ *mrTRG*, MRI-based tumor regression grading, followed the definition from MERCURY study group.

**Supplemental Table 7.** **Comparison of accuracy between MR images and histopathological findings in three approaches**

| **MR images** | **Pathologic findings** | | |
| --- | --- | --- | --- |
|  | ypT stage  (*n* = 15) | pCR ^†^  (*n* = 7) | near-pCR ^#^  (*n* = 8) |
| In vivo 3.0T | 8/15 | 1/7 | 6/8 ^*^ |
| Ex vivo 3.0T | 12/15 | 7/7 | 8/8 |
| Ex vivo 9.4T | 15/15 | 7/7 | 8/8 |
| *p* value | 0.0086 | 0.0002 | 0.1128 |

^†^ *pCR*, identified as pTRG=0 (ypT0N0M0),

^#^ *near-pCR*, identified as pTRG=0-1,

^*^ identified as mrTRG1-2

**
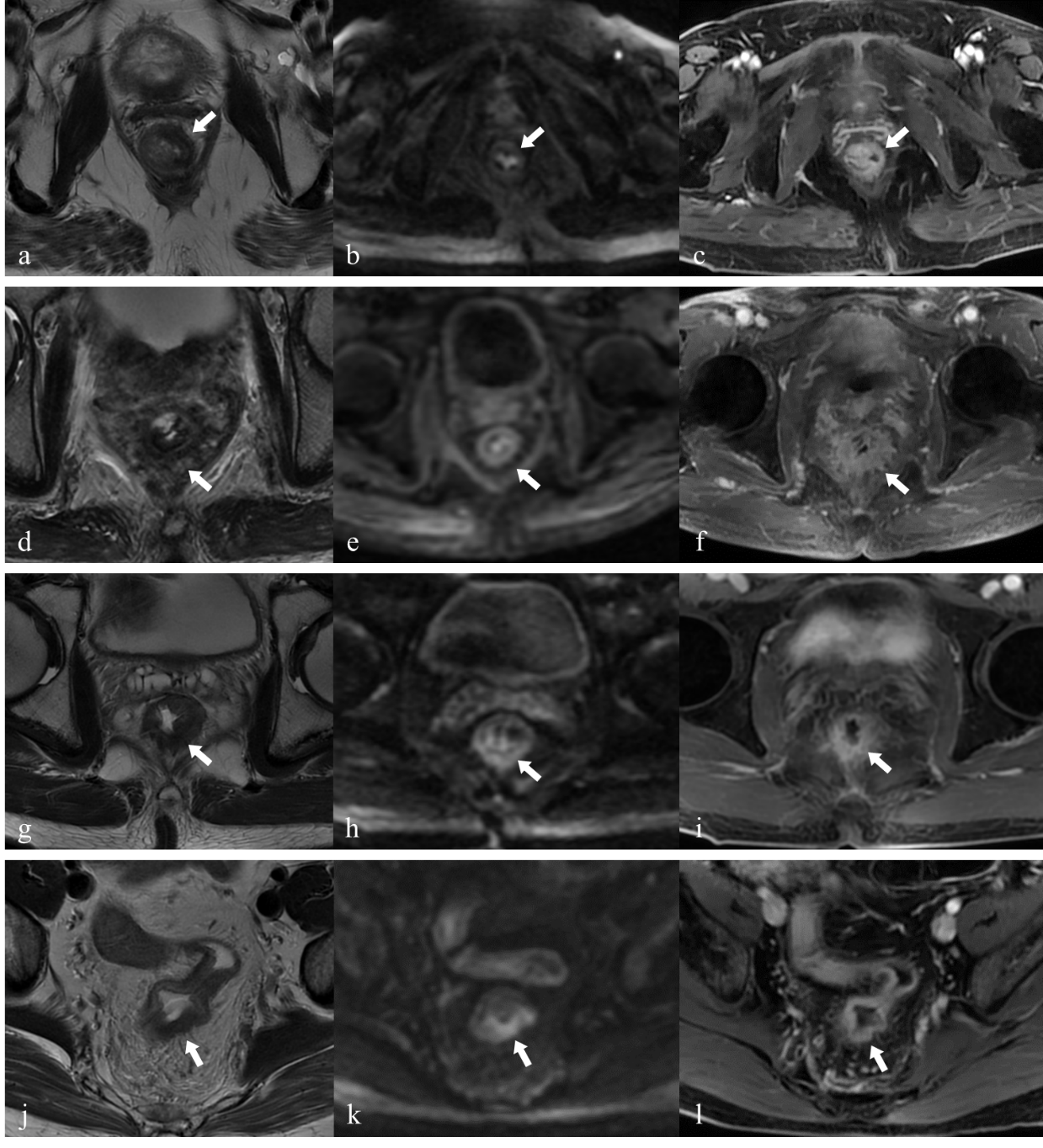
**

**Supplemental Figure 1**. Preoperative images of in vivo MRI for evaluating mrTRG obtained from RC patients who received nCRT. (a-c) high-resolution T2WI, DWI and CE-T1WI of one case of pTRG 0 was diagnosed as mrTRG 2 on in vivo MRI. (d-f) high-resolution T2WI, DWI and CE-T1WI of one case of pTRG 1 was recognized as mrTRG 2 on in vivo MR scan. (g-i) high-resolution T2WI, DWI and CE-T1WI of one case of pTRG 2 was diagnosed as mrTRG 3 on in vivo MR scan. (j-l) high-resolution T2WI, DWI and CE-T1WI of one case of pTRG 3 was diagnosed as mrTRG 3 on in vivo MR scan.


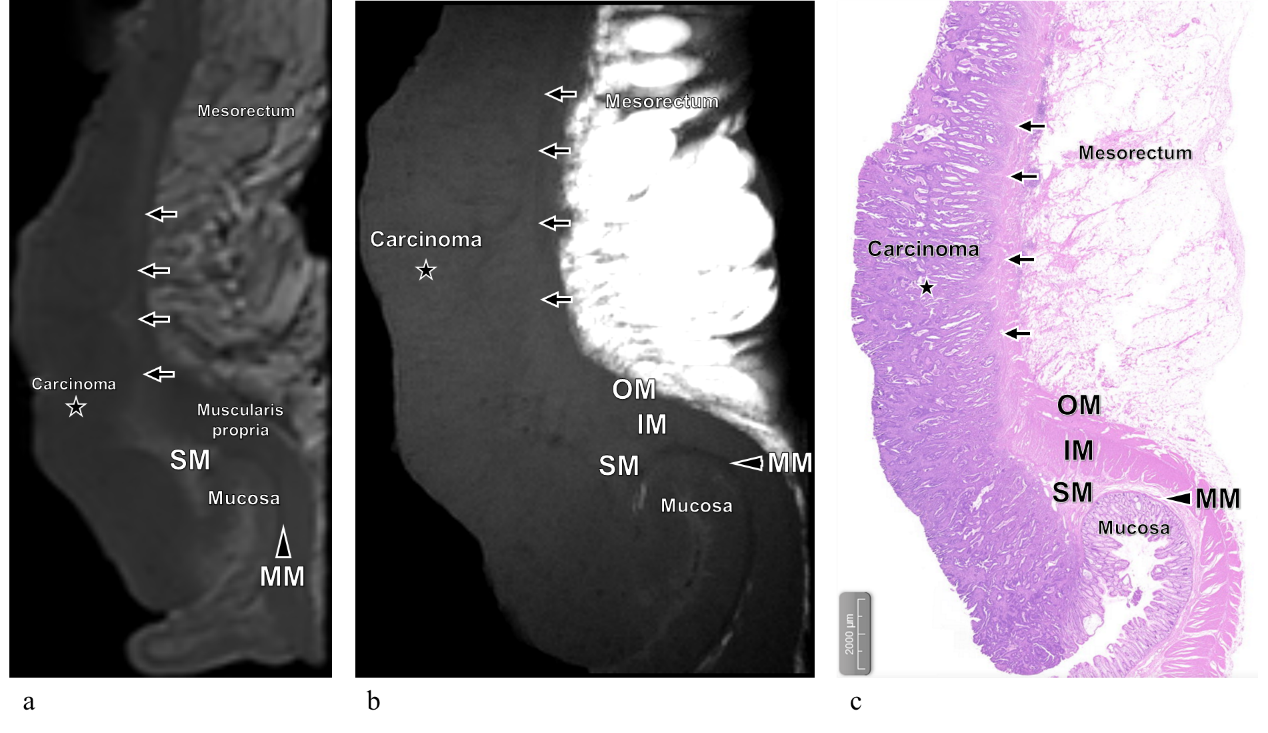


**Supplemental Figure 2**. Postoperative images of the rectal adenocarcinoma specimen without nCRT. (a) Ex vivo T2-weighted 3.0T MR image and (b) ex vivo T2-weighted 9.4T MR image clearly show that an irregularly shaped tumor (star) has partially invaded the muscularis propria layer (arrows) and unclear hierarchical structure between inner and outer muscle layers without no invasion to the mesorectum, diagnosed as T2. (c) Corresponding histopathologic slice shows carcinoma (star) has involved the muscularis propria (arrows). (Hematoxylin-eosin stain; original magnification, ×10.)
